# Moxibustion for primary dysmenorrhea: A resting-state functional magnetic resonance imaging study exploring the alteration of functional connectivity strength and functional connectivity

**DOI:** 10.3389/fnins.2022.969064

**Published:** 2022-08-30

**Authors:** Han Yang, Xiang Li, Xiao-li Guo, Jun Zhou, Zhi-fu Shen, Li-ying Liu, Wei Wei, Lu Yang, Zheng Yu, Jiao Chen, Fan-rong Liang, Si-yi Yu, Jie Yang

**Affiliations:** ^1^Acupuncture and Tuina School, Chengdu University of Traditional Chinese Medicine, Chengdu, China; ^2^Chengdu Xi’nan Gynecological Hospital, Chengdu, China; ^3^Department of Traditional Chinese and Western Medicine, North Sichuan Medical College, Nanchong, China; ^4^College of Medical Information and Engineering, Chengdu University of Traditional Chinese Medicine, Chengdu, China; ^5^Acupuncture & Brain Research Center, Chengdu University of Traditional Chinese Medicine, Chengdu, China

**Keywords:** moxibustion, primary dysmenorrhea, functional connectivity strength, default mode network, functional connectivity, left inferior frontal gyrus

## Abstract

**Introduction:**

Primary dysmenorrhea (PDM) is a common gynecological disease and chronic pain disorder. Moxibustion, a form of traditional Chinese medicine therapy, has proven to be effective for PDM. However, the central mechanisms of PDM and moxibustion for PDM are still unclear. This study aims to explore the potential central mechanism of PDM and clarify the possible mechanism of moxibustion for relieving pain.

**Materials and methods:**

A total of 23 PDM patients and 23 matched healthy controls (HCs) were enrolled. For PDM patients, resting-state functional magnetic resonance imaging (rs-fMRI) data were collected pre- and post-moxibustion treatment of 3 consecutive menstrual cycles, respectively. For HCs, rs-fMRI data were collected in the baseline. The resting-state functional connectivity strength (rs-FCS) analysis and the resting-state functional connectivity (rs-FC) analysis based on the region of interest (ROI) were combined to be conducted.

**Results:**

Compared to HCs, PDM patients showed weaker rs-FCS in the left inferior frontal gyrus (IFG). After the moxibustion treatment, rs-FCS in the left IFG was increased with clinical improvement. Then, the left IFG was chosen as ROI, and the rs-FC analysis was conducted. It showed that the left IFG rs-FC in the bilateral anterior cingulate cortex (ACC)/middle cingulate cortex (MCC), the left posterior cingulate cortex (PCC)/precuneus (PCU), and the left parahippocampal gyrus (PHG) decreased after moxibustion treatment, most of which belong to the default mode network (DMN).

**Conclusion:**

Our results highlight the role of the left IFG and the DMN in PDM. Specifically, the central mechanism of moxibustion for analgesia may be related to modulating the disorders of the reappraisal and processing of pain stimuli through influencing the cognition of pain.

## Introduction

Primary dysmenorrhea (PDM) is one of the most general gynecological diseases, widely affecting adolescent girls and women of reproductive age (Burnett and Lemyre, 2017). It is mainly manifested as colic pain in the lower abdomen during menstruation without any identified pathological conditions (Iacovides et al., 2015) and is sometimes associated with other symptoms, including headaches, fatigue, diarrhea, vomiting, and sweating (Zhang et al., 2020, 2022). According to the surveys, at least one-third of women are reported to have experienced moderate or severe menstrual pain, which affects their daily life and causes considerable social and economic losses (Kennedy, 1997; Yang et al., 2017; Yesuf et al., 2018).

At present, some pharmacological and non-pharmacological methods are chosen to treat PDM. Among the pharmacological treatments, non-steroidal anti-inflammatory drugs (NSAIDs) and oral contraceptives are commonly used (Dawood, 2006). It is reported that NSAIDs are effective in relieving symptoms of patients with PDM (Zahradnik et al., 2010), while they are often accompanied by various side effects, such as nephrotoxic and hepatotoxic effects, gastrointestinal discomfort, and fluid retention (Dawood, 2006). Besides, for about 20% of women, NSAIDs have no positive effect on alleviating menstrual pain (Proctor and Murphy, 2001). For patients who can be relieved of immediate pain, NSAIDs cannot further prevent the progression of PDM (Yang et al., 2017). As a result, it is necessary to search for a non-pharmacological treatment that is effective and safe for PDM.

Moxibustion, one of the treatments of traditional Chinese medicine, is widely used for gynecological disorders in China. A systematic review shows that moxibustion is effective for PDM (Gou et al., 2016). In addition, the follow-up effectiveness of moxibustion has been proved to be better compared to NSAIDs (Yang et al., 2017). A well-recognized pathophysiology of PDM is an increase in prostaglandins, which leads to uterine contraction, thus limiting blood flow and leading to cramps (Ferries-Rowe et al., 2020). In addition, the state of long-term recurrent pain is thought to affect the central nervous system, resulting in pathological changes in the structure and function of the brain (Dun et al., 2017; Yang et al., 2019; Quan et al., 2021), further aggravating PDM (Zhang et al., 2022). Prior work (Gou et al., 2016; Yang et al., 2017) has found that moxibustion can lower the prostaglandin F_2α_ (PGF_2α_) level in serum, then relieving the contraction of uterine contraction, ultimately reducing pain. However, the central mechanism of moxibustion for PDM remains limited.

Resting-state functional magnetic resonance imaging (rs-fMRI) technology has been widely used in pain and analgesia research (Baumbach et al., 2022; Li et al., 2022; Wei et al., 2022). This technology can measure continuous spontaneous brain activity and draw interregional functional connectivity (FC) (Biswal et al., 1995; Fox and Raichle, 2007). Functional connectivity strength (FCS) is a data-driven method based on the voxel level of the whole brain. It can evaluate the internal function of the brain by identifying the brain sub-network (Liang et al., 2013; Zuo et al., 2019). The FC analysis can analyze the strength of the FC relationship between one specific brain region and other brain regions from the perspective of functional integration (Zuo et al., 2019). The combination of the two methods is helpful to break the excessive dependence on *a priori* information and comprehensively exploring the change of brain functional activities related to disease and intervention (Yang et al., 2018), which has been used frequently in recent years (Ke et al., 2020; Cao et al., 2022).

The purpose of this study is to explore the potential central mechanism of PDM and clarify the possible mechanism of moxibustion in treating PDM. Therefore, the resting-state functional connectivity strength (rs-FCS) method was adopted to compare the difference between PDM patients and healthy controls (HCs) based on the data-driven approach and the rs-FCS of the voxel level was calculated to evaluate whether moxibustion can normalize the abnormal functional changes in the brain regions in PDM patients. The overlapped brain region was defined as that showing a significant difference in rs-FCS both between the PDM patients and HCs and between the pre- and post-moxibustion of PDM patients. Then, the most relevant overlapped brain region was chosen as the region of interest (ROI) to conduct the resting-state functional connectivity (rs-FC) analysis to compare the change between the pre- and post-moxibustion to further explore the possible influence of moxibustion. We hypothesized that (1) PDM patients had dysfunction in the brain regions related to processing pain stimuli, and (2) effective treatment might regulate the dysfunction of the relevant brain areas.

## Materials and methods

### Participants

We recruited 23 PDM patients and 23 female HCs from 1 March 2013 to 12 August 2013. All the participants were from universities in Chengdu, Sichuan, China. Our study has been ethically reviewed and approved by the Sichuan Regional Ethics Review Committee on Traditional Chinese Medicine (2013KL-004). Before the start of the trial, all participants signed written informed consent and received a pelvic B-mode ultrasonography exam by gynecologists to exclude organic pelvic disease. Based on the diagnostic standards of the Clinical Guideline of Primary Dysmenorrhea by the Society of Obstetricians and Gynecologists of Canada (Lefebvre et al., 2005), the PDM patients were considered eligible if they met the following criteria: (Burnett and Lemyre, 2017) 18 ∼ 35 years old; (Iacovides et al., 2015) having regular menstrual cycles (28 days ± 7 days); (Zhang et al., 2022) average menstrual pain score in the past 3 months was no less than 4, measured by a visual analog scale (VAS) before the start of the trial; (Zhang et al., 2020) right-handedness; and (Kennedy, 1997) handwriting informed consent form. The inclusion criteria for HCs were the same as those of the PDM patients except that they had not experienced menstrual pain. For both the PDM patients and HCs, the exclusion criteria were as follows: (Burnett and Lemyre, 2017) secondary dysmenorrhea caused by organic pelvic disease; (Iacovides et al., 2015) having irregular menstrual cycles; (Zhang et al., 2022) suffering from uncontrolled nervous system diseases, immune deficiency, hemorrhagic diseases, mental disorders, and allergies; (Zhang et al., 2020) being pregnant, or lactating, or with pregnancy plans within half a year; (Kennedy, 1997) taking prostaglandin synthetase inhibitor 2 weeks before enrollment; (Yang et al., 2017) receiving any treatment 2 weeks before enrollment; (Yesuf et al., 2018) taking medicine which may influence outcomes; (Dawood, 2006) having any contraindications to magnetic resonance imaging (MRI); and (Zahradnik et al., 2010) undergoing other trials. Based on the above 9 items, the PDM patients were also excluded if they met contraindications to moxibustion (i.e., skin lesions at the selected acupoints and moxa intolerance). All the participants were not allowed to consume alcohol or caffeine within 24 h before the MRI scan.

### Intervention

All the enrolled PDM patients were treated with moxibustion for 3 consecutive menstrual cycles for a total of 3 sessions. Each session of the treatment started 7 days before the beginning of menses and did not cease until menstruation occurred, about 30 min per time, once a day, 7 times a session. Considering that in the traditional Chinese medicine theory, Guanyuan (CV4), Shenque (CV8), and Sanyinjiao (SP6) were the well-known acupoints for treating gynecological diseases (Liao et al., 2019), they were selected for our study. The lighting moxa roll (Z32021062, Oriental Moxa Co., Suzhou, China) was placed approximately 2 ∼ 3 cm away from the skin surface of Guanyuan (CV4) and Shenque (CV8) simultaneously for about 15 min, and then, moved to bilateral Sanyinjiao (SP6) at the same time also for about 15 min, with circling at an even speed to stimulate by a comfortable moxa heat feeling, which was similar to the sensation of “Deqi” in acupuncture.

### Clinical and laboratory outcome measurements

The clinical outcomes were (Burnett and Lemyre, 2017) pain intensity measured by VAS; (Iacovides et al., 2015) menstrual symptom intensity and duration scores measured by Cox Menstrual Symptom Scale (CMSS). The laboratory outcomes were PGF_2α_ and oxytocin (OT). These data were only collected in the PDM patients and all outcomes were measured pre- and post-moxibustion treatment.

### Magnetic resonance imaging data acquisition

All participants underwent MRI scans on a 3.0T scanner (Allegra; Siemens Medical System, Erlangen, Germany) with an eight-channel head coil at the Department of Radiology at the Huaxi MR Research Center, West China Hospital of Sichuan University, during the ovulatory period (days 10 ∼ 14 of the menstrual cycle). Among them, the PDM patients underwent MRI scans in the latest ovulatory period pre- and post-treatment, and the HCs only underwent MRI scans at baseline. The participants were asked to stay awake during the scan (eyes closed, head stationary but relaxed, and thinking about nothing). Head cushions were chosen to reduce head movement and earplugs were used to minimize noise.

The scan parameters were as follows: T1-weighted images: repetition time = 8.16 ms, echo time = 3.18 ms, flip angle = 7°, field of view = 256 × 256 mm, matrix = 256 × 256; slice thickness = 1 mm, no gap; and 188 sagittal slices; rs-fMRI were obtained in 7 min with a gradient-recalled echo-planar imaging pulse sequence: repetition time = 2,000 ms, echo time = 30 ms, flip angle = 90°, acquisition matrix = 64 × 64, field of view = 240 × 240 mm, thickness = 4.0 mm, voxel size = 3.5 × 3.5 × 4.02 mm^3^, gap = 0.5 mm, NEX = 1.0, number of slices = 33. A total of 210 volumes were acquired.

### Data preprocessing

The image data were analyzed by the Data Processing Assistant for Resting-State fMRI (DPARSF), based on the Statistical Parameter Mapping software (SPM12^1^) in MATLAB 2014b (The MathWorks, Inc., Natick, MA, United States). Considering the initial instability of the MRI signal, the first 10 time points were abandoned. In addition, other preprocessing was also operated, including slice timing, realignment for head motion correction, co-registration of T1-weighted anatomical images for each participant, and normalization against the Montreal Neurological Institute template. Participants with head movements greater than 2° or 2 mm were excluded rather than further analyzed. When normalized to the Montreal Neurological Institute space, the images were resliced with isotropic 3 mm^3^ voxel size, and spatial smoothing was performed by using a Gaussian kernel with full width at half-maximum (FWHM) of 6 mm. After removing the linear trends, the images were filtered with a temporal bandpass of 0.01 ∼ 0.08 Hz. Covariates related to white matter noise, cerebrospinal fluid, and head movement were regressed. Finally, several sources of spurious variances were removed by linear regression, including six head motion parameters, as well as the average signals of cerebrospinal fluid, white matter, and whole brain.

### Statistical analysis

#### Clinical and laboratory data analysis

SPSS 26.0 software (SPSS Inc., Chicago, IL, United States) was adopted to analyze the clinical and laboratory data. A threshold was set at *p* < 0.05 (2-tailed) According to whether the data met the normal distribution, *T*-test and chi-square test were selected to compare the baseline data between groups and the difference in assessment pre- and post-treatment in the PDM patients.

#### Functional connectivity strength analysis

Whole-brain rs-FC analysis was conducted according to the following procedure. First, to eliminate the artifactual correlation in the non-gray matter, the average value of the gray matter (GM) probability map of all participants was extracted through the threshold (cutoff = 0.2) to generate the GM mask. Then, the time series of each voxel within the GM mask was extracted, and Pearson’s correlations between any pair of voxels of each participant were computed. For improving the normality, Fisher’s r-to-z transformation was chosen to convert the FC matrix to a z-score. Subsequently, nodal FCS as the sum of weights connected with other voxels for a given voxel (node) was calculated. To eliminate voxels with weak correlation due to signal noise, the computation was restricted to connections with the absolute value of the correlation coefficient above 0.2 conservatively (Li et al., 2015).

A two-sample *t*-test was chosen to compare the different rs-FCS in the whole brain between the PDM patients and HCs in the baseline. Paired *t*-test was chosen to compare the different rs-FCS in the whole brain pre- and post-moxibustion treatment in the PDM patients. The overlapped brain region was determined as showing different rs-FCS both between PDM patients and HCs, and between pre- and postmoxibustion treatment in the PDM patients.

#### Functional connectivity analysis

We chose the overlapped brain region with different rs-FCS as the ROI to further conduct the rs-FC analysis. To improve the normality, we selected Fisher’s r-to-z transformation to convert the individual correlation matrix into a z-score matrix. Each PDM patient was generated the FC map based on the ROI. To compare the effect of moxibustion on PDM, paired *t*-test was chosen to evaluate the changes of rs-FC pre- and post-moxibustion treatment. According to previous studies (Bansal and Peterson, 2018), cluster–forming voxel-wise threshold (*Z* > 2.5) and a corrected cluster significance threshold (*p* < 0.05) were used for multiple comparisons.

## Results

A total of 46 participants were enrolled in our study, including 23 PDM patients and 23 HCs. Among the 23 PDM patients, 2 patients dropped out because of schedule conflicts. In addition, the other 3 patients did not complete the laboratory data collection pre- and post-moxibustion treatment. Finally, the data of a total of 23 patients and 23 HCs were compared at baseline, and the data of 21 patients were compared pre- and post-moxibustion treatment (among them, 18 patients were compared in laboratory data).

### Demographics and clinical characteristics

The baseline data of all participants are shown in Table 1. There were no significant differences between the PDM patients and HCs in age, height, and weight (*p* > 0.05). For the baseline characteristics in the PDM patients, the days of dysmenorrhea, unable to work, and accompanying symptoms in the past 3 months were 4.00 (3.00, 7.00), 2.00 (1.00, 3.00), and 3.00 (3.00, 7.00), respectively.

**TABLE 1:** Baseline participant characteristics.

		Group		t	*P-value* *
				t	
		PDM group (*n* = 23)	HC group (*n* = 23)		
	Age (years)	21.74 ± 2.01	22.26 ± 2.12	–0.86	0.395
	Height (cm)	160.65 ± 4.10	158.52 ± 5.35	1.52	0.137
	Weight (kg)	50.04 ± 4.57	51.78 ± 9.37	–0.8	0.43
Days in the Past 3 Months	Dysmenorrhea	4.00 (3.00, 7.00)	/	/	/
	Unable to Work	2.00 (1.00, 3.00)	/	/	/
	Accompanying symptoms	3.00 (3.00, 7.00)	/	/	/

Data (age, height, and weight) was given as mean ± standard deviation.

Data (days of dysmenorrhea in the past 3 months, days unable to work in the past 3 months, and days with accompanying symptoms in the past 3 months) was given as Q_50_ (Q_25_, Q_75_). *p < 0.05 was considered statistically significant.

### Clinical and laboratory characteristics of pre- and post-moxibustion

The clinical and laboratory data of pre- and post-moxibustion in the PDM group are shown in Table 2. The result of paired chi-square test suggested a significant difference between pre- and post-moxibustion in the PDM patients with VAS scores [pre: 6.90 (6.40, 7.35), post: 2.90 (2.15, 3.40), *p* < 0.001], which showed moderate to severe menstrual pain before moxibustion and slight menstrual pain after moxibustion. In addition, it also showed a significant difference between pre- and post-moxibustion in the menstrual symptom intensity scores measured by CMSS and menstrual symptom duration scores measured by CMSS, PGF_2α_ , and OT (*p* < 0.001).

**TABLE 2:** Clinical and laboratory outcomes of pre- and post- moxibustion in PDM group.

	PDM-pre	PDM-post	Z	P
Pain intensity measured by VAS	6.90 (6.40, 7.35)	2.90 (2.15, 3.40)	–4.016	< 0.001
Menstrual symptom intensity scores measured by CMSS	19.00 (13.50, 26.50)	5.00 (3.00, 8.00)	–4.019	< 0.001
Menstrual symptom duration scores measured by CMSS (days)	29.00 (18.00, 35.00)	6.00 (3.00, 7.50)	–4.016	< 0.001
PGF_2α_	689.46 (656.31, 784.05)	295.19 (247.17, 362.23)	–3.724	< 0.001
OT	155.84 (146.23, 162.89)	74.52 (49.16, 84.08)	–3.724	<0.001

Data was given as Q_50_ (Q_25_, Q_75_).

Data of 21 PDM patients pre- and post- moxibustion was analyzed in pain intensity measured by VAS, menstrual symptom intensity scores measured by CMSS, and menstrual symptom duration scores measured by CMSS.

Data of 18 PDM patients pre- and post- moxibustion was analyzed in PGF_2α_ and OT.

### Resting-state functional connectivity strength results

**FIGURE 1:**
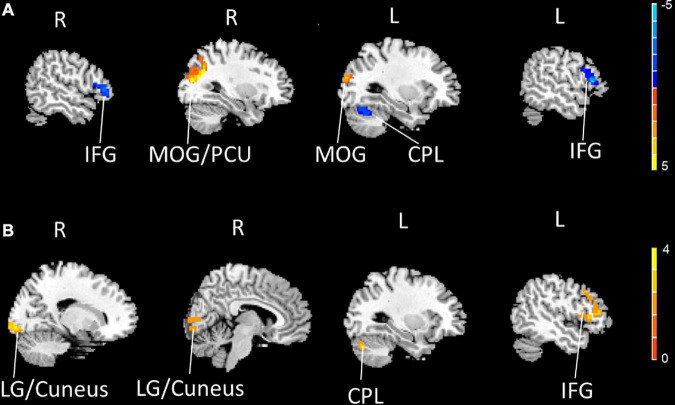
The bilateral IFG and the left CPL showed decreased rs-FCS (blue), and the bilateral MOG and the right PCU showed increased rs-FCS (orange-yellow) in PDM patients compared to HCs (voxel level *p* < 0.005, cluster-level *p* < 0.05, a cluster-forming threshold of 2.5) **(A)**. The left IFG, the left CPL and the right LG/cuneus showed increased rs-FCS (orange-yellow) post-moxibustion compared to pre-moxibustion in PDM patients (voxel level *p* < 0.005, cluster-level *p* < 0.05, a cluster-forming threshold of 2.5) **(B)**. IFG, inferior frontal gyrus; CPL, cerebellum posterior lobe; rs-FCS, resting-state functional connectivity strength; MOG, middle occipital gyrus; PCU, precuneus; PDM, primary dysmenorrhea; HC, healthy control; LG, lingual gyrus.

Compared to the HCs, the PDM patients exhibited weaker rs-FCS in the bilateral inferior frontal gyrus (IFG), the left cerebellum posterior lobe (CPL), and stronger rs-FCS in the bilateral middle occipital gyrus (MOG) and the right precuneus (PCU) after controlling for age (Figure 1A and Table 3).

**TABLE 3:** Regions showing significantly different rs-FCS in PDM patients and HCs, before and after 3-month intervention.

Brain region	BA	Voxel size	MNI coordinates (RAI)			Peak *Z* Score
				
			x	y	z	
**PDM (*n* = 23) VS HC (*n* = 23)**						
Left IFG	45	97	–51	27	9	–5.40
Left CPL	/	334	–21	–51	–21	–3.88
Right MOG/PCU	19	191	30	-66	30	4.55
Left MOG	19	98	–36	–87	12	3.19
Right IFG	45	91	51	36	9	–3.88
**PDM-post (*n* = 21) VS PDM-pre (*n* = 21)**						
Left IFG	45	122	–54	30	12	4.69
Left IFG	45	53	–48	21	39	3.14
Right LG/Cuneus	17	123	18	–93	–6	3.96
Left CPL	/	50	–27	-75	–30	3.28

IFG, inferior frontal gyrus; CPL, cerebellum posterior lobe; MOG, middle occipital gyrus; PCU, precuneus; LG, lingual gyrus.

**FIGURE 2:**
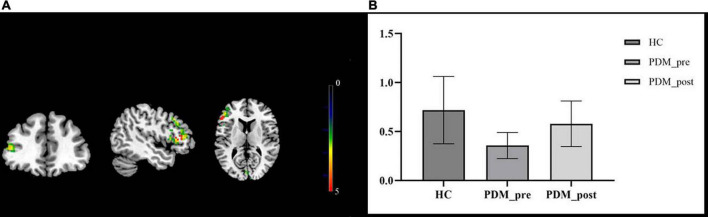
The overlapped brain region—the left IFG. The rs-FCS of the left IFG in PDM patients showed decreased (green) when compared with HCs’ and increased (red) after moxibustion treatment. The overlapped brain region was marked as yellow **(A)**. Rs-FCS value of the left IFG in different conditions. Compared to the HCs, PDM patients exhibited weaker rs-FCS in the left IFG and it showed to be normalized after moxibustion treatment **(B)**. IFG, inferior frontal gyrus; rs-FCS, resting-state functional connectivity strength; HC, healthy control; PDM, primary dysmenorrhea.

We then compared the post- and pre-moxibustion differences in the PDM group. The results revealed that after moxibustion treatment, the PDM patients showed increased rs-FCS in the left IFG, the CPL, and the right lingual gyrus (LG)/cuneus (Figure 1B and Table 3). Interestingly, this longitudinal finding partially overlapped with the rs-FCS difference in the left IFG between HCs and PDM patients at baseline (Figure 2A). The rs-FCS value of the left IFG in different conditions is shown in Figure 2B.

### Left inferior frontal gyrus resting-state functional connectivity results

**FIGURE 3:**
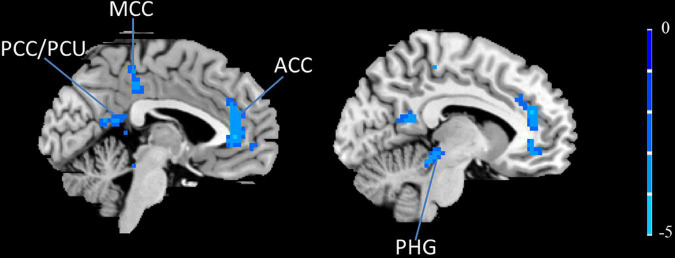
The left IFG rs-FC decreased (blue) in the bilateral ACC, the bilateral MCC, the left PCC/PCU and the left PHG in PDM patients after moxibustion treatment. IFG, inferior frontal gyrus; rs-FC, resting-state functional connectivity; ACC, anterior cingulate cortex; MCC, middle cingulate cortex; PCC, posterior cingulate cortex; PCU, precuneus; PHG, parahippocampal gyrus; PDM, primary dysmenorrhea.

To further explore the influence of moxibustion on the brain’s functional activities, the left IFG was chosen as the ROI to conduct the rs-FC analysis. The results showed that the left IFG rs-FC in the bilateral anterior cingulate cortex (ACC)/the middle cingulate cortex (MCC), the left posterior cingulate cortex (PCC)/PCU, and the left parahippocampal gyrus (PHG) decreased in the PDM patients after moxibustion treatment (Figure 3 and Table 4).

**TABLE 4:** Regions showing significantly different of the left IFG rs-FC in PDM patients before and after 3-month intervention.

Brain region	BA	Voxel size	MNI coordinates (RAI)			Peak *Z* Score
			x	y	z	
Bilateral ACC	32	247	**–**6	39	21	**–**5.65
Left PHG	/	70	**-**12	**–**33	**–**9	**–**5.26
Left PCC/PCU	29/30	63	**–**6	**–**57	15	**–**3.97
Bilateral MCC	31	62	0	**–**36	31	**–**4.34

Data of 21 PDM patients pre- and post- moxibustion was analyzed.

ACC, anterior cingulate cortex; PHG, parahippocampal gyrus; PCC, posterior cingulate cortex; PCU, pre-cuneus; MCC, middle cingulate cortex.

## Discussion

This study, for the first time, investigated the potential central mechanism of moxibustion for PDM. We focused on the differences in rs-FCS between the PDM patients and HCs, as well as the changes of rs-FCS in the PDM patients pre- and post-moxibustion treatment. Compared to HCs, the rs-FCS of the left IFG in the PDM patients was found to be decreased significantly. After moxibustion, the rs-FCS of the left IFG in the PDM patients increased (normalized) significantly. Then, the left IFG was identified as the key overlapped brain region to conduct the rs-FC analysis in the PDM patients and its rs-FC with the bilateral ACC/MCC, the left PCC/PCU, and the left PHG was found to be decreased after moxibustion.

Moxibustion has a long history of being used to treat PDM in China and has been proven effective in previous studies (Li et al., 2006; She et al., 2008; Yang et al., 2008; Sun et al., 2009; Zhu et al., 2010). In our study, after 3 menstrual cycles of moxibustion treatment, the values of clinical outcomes (pain intensity measured by VAS, and menstrual symptom intensity and duration scores measured by CMSS) and laboratory outcomes (PGF_2α_ and OT) decreased significantly, which showed a consistent effect with previous studies (Wang et al., 2005). Considering the effect of moxibustion in PDM, it is particularly important to explore the central mechanism to determine the key target for the diagnosis and treatment of PDM.

Our results showed that decreased rs-FCS of the left IFG in PDM patients had increased (normalized) after moxibustion. Previous neuroimaging studies have suggested that IFG was related to chronic pain (Ning et al., 2018). The left IFG, which is reportedly involved in executive cognitive function and episodic memory, belongs to the executive control network (ECN) (Xue et al., 2021). In terms of cognition, the IFG cortex participates in processing emotions in the human brain (Boulloche et al., 2010), such as fear related to pain (Biggs et al., 2020). Pain was thought to cause negative emotion and the negative emotion is thought to, in turn, aggravate the feeling of pain and form a vicious circle (Bushnell et al., 2013).

Previous research has shown that PDM patients have structural and functional abnormalities in the DMN (Liu et al., 2017). In our study, the left IFG was chosen as ROI to conduct the rs-FC analysis. Interestingly, we found that most of these brain regions (i.e., ACC, MCC, and left PCC/PCU) with significant changes after moxibustion treatment belong to the DMN.

The DMN refers to the areas with relatively active functional activities in the midline and lateral cortex of the brain in the resting state. These brain areas have spontaneous activation, time synchronization, and internal FC. The DMN is the neural basis of many important functional activities of humans, including cognition, consciousness, etc. (Hu et al., 2022; Oyarzabal et al., 2022; Wang et al., 2022) It participates in a lot of neurophysiological processes, such as the evaluation of episodic memory, introspection, monitoring of the external environment, emotional processing, and cognitive control (Greicius et al., 2003; Arsenault et al., 2018; Li J. et al., 2021; Oyarzabal et al., 2022; Tanglay et al., 2022). Studies have confirmed that in the state of chronic pain, the activation-inactivation mechanics of the DMN is in disorder (Baliki et al., 2008; Shen et al., 2019). It is the main resting state network affected by chronic pain (Baliki et al., 2008), and it is also the network affected at first and most obviously by chronic pain (Baliki et al., 2008; Cauda et al., 2009). Although the exact function of the DMN has not been fully determined, many studies have found that the FC between the DMN and other pain-processing brain areas was enhanced in populations with varied pain syndromes, such as diabetic neuropathic pain and chronic back pain (Baliki et al., 2008; Cauda et al., 2009), which indicates that pain destroys the DMN by increasing the nociceptive stimulus input.

A recent study reported that pain cueing to personal potential discomfort led to more attention and was subsequently identified more accurately (Zuo et al., 2022). It suggests that women who have experienced menstrual pain, especially if they are suffering from menstrual pain, will pay more attention to the painful period when they are about to have their period, due to the unpleasant memories of their previous periods. In our study, the effect of moxibustion may be closely related to mediating the disorders of attentional modulations in the DMN of PDM patients. The unusually close interplay between attention and pain seems to involve pain-specific features (Wiech et al., 2008). Studies suggested that attention has the ability in modulating sensory and affective levels of pain, which was possible to be regulated by integrating pain spatially (Miron et al., 1989; Villemure et al., 2003; Quevedo and Coghill, 2007). The ACC, one of the areas of the DMN, was shown to modulate attention (Wiech et al., 2008). The activity of the dorsal ACC is related to the critical point between approach and avoidance behavior (Schlund et al., 2016). Besides, a particularly interesting point is that avoidance behavior is important in chronic pain disability maintenance. As a result, some scholars suggest that the dorsal ACC is a key area associated with pain-related fear responses. Moxibustion may affect the pain-related fear by affecting the ACC, which may be responsible for modulating attention disorder of pain. The area MCC in the DMN has also been reported to be involved in modulating the affective-motivational dimension to influence attention to pain (Liang et al., 2022). It is an area rich in dopaminergic innervation, which is thought to be activated in response to noxious stimuli (Liang et al., 2022). There was an abnormal increase of rs-FC between the prefrontal cortex and the MCC of patients with chronic low back pain patients reported in a previous study (Mao et al., 2022), which hinted at the connection between the MCC and pain. The other significantly changed areas after treatment, the PCC and the PCU, are regarded to play vital roles in the DMN (Greicius et al., 2003). They are involved not only in sensory processing but also in the extraction of episodic memory (Maguire and Mummery, 1999; Maddock et al., 2001; Fujii et al., 2002). It has been reported that women with prolonged menstrual pain might tend to pay more attention to pain, which can lead to abnormal activity of the PCC and the PCU (Dun et al., 2020). Our results showed that moxibustion might affect the left PHG apart from the above DMN brain regions in the PDM patients. The left PHG is thought to be associated with memory in humans (Saykin et al., 1999). The decreased left IFG rs-FC in the left PHG may influence memory related to menstrual pain.

Both attention and memory are important parts of cognition. In chronic pain, cognitive modulation is considered of particular significance (Bishop, 2007), which can regulate the perception of pain (Wiech et al., 2008). The IFG and the core brain areas in the DMN are central areas in the predictive and reactive control systems (PARCS). PARCS, which consists of the reactive system and the predictive system, controls response to a novel, salient, and urgent stimuli and focuses attention on stimuli that are urgent and close in time and space. Due to the functional hemispheric asymmetry of the IFG, the right IFG involves the appraisal of the stimulus and the left IFG involves the reappraisal of the stimulus based on a pre-existing internal model. The left IFG plays an important role in the assimilation of new and emotional information into the existing internal model, and thus, has an important mediating role between the reactive and predictive control (Tops et al., 2014).

Considering the DMN as a part of the predictive system, alterations in the rs-FC between the left IFG and the DMN may have contributed to the change of reappraisal and processing of menstrual pain stimuli, which may be through influencing the cognition of pain (Li F. et al., 2021). Neuroimaging studies suggested that abnormal FC might be an important factor in pain disorders (Li F. et al., 2021). It reflected the effect of one brain region or brain network on another, thus indicating a causal relationship between different brain regions or brain networks (Friston, 1994). In a previous study, a reciprocal inhibitory effect between the ECN and the DMN was found and confirmed that the anti-correlation between them persisted in their causal relationship (Uddin et al., 2009). Besides, it has been found that migraine may be related to abnormal pattern connectivity of the ECN and the DMN (Zou et al., 2021). In consideration of the left IFG being one of the regions of the ECN, our study suggests that moxibustion may affect the rs-FC between the ECN and the DMN.

Taken together, we suggest that PDM may be mainly caused by abnormal functional alteration of the left IFG and moxibustion tend to normalize it. In addition, moxibustion may work by affecting the rs-FC between the left IFG and the DMN to modulate the disorders of the reappraisal and processing of pain stimuli in PDM patients to realize clinical improvement.

However, due to the lack of PDM patients without moxibustion treatment as a control, randomized controlled trials will be needed to further explore the neuroimaging mechanisms, such as setting moxibustion group versus waiting group, or moxibustion group versus acupuncture group. In addition, the study was only based on the functional image. The multimodality images will be expected in the future to illustrate the central mechanism from multiple dimensions. Further, we did not collect the follow-up fMRI data to determine the long-term influence of moxibustion on functional brain activity, which should be considered in future study designs when funding is enough.

## Conclusion

In sum, our results highlight the role of the left IFG and the DMN in PDM. The central mechanism of moxibustion for analgesia may be related to modulating the disorders of the reappraisal and processing of pain stimuli through influencing the cognition of pain. Such findings have implications for understanding the pathophysiology and treatment of PMD and may help in clinical practice and further research in the future.

## Data availability statement

The raw data supporting the conclusions of this article will be made available by the authors, without undue reservation.

## Ethics statement

The studies involving human participants were reviewed and approved by Sichuan Regional Ethics Review Committee on Traditional Chinese Medicine. The patients/participants provided their written informed consent to participate in this study. Written informed consent was obtained from the individual(s) for the publication of any potentially identifiable images or data included in this article.

## Author contributions

F-RL, and JY study conceptualization. S-YY, JC, ZY, and XL study design. Z-FS, WW, X-LG, L-YL, and LY data collection. S-YY, HY, and JZ data analysis. HY, S-YY, and XL manuscript preparation. All authors read and approved the final manuscript.

## Footnotes

1
http://www.fil.ion.ucl.ac.uk/spm

## Abbreviations

ACC: anterior cingulate cortex
CMSS: cox menstrual symptom scale
CPL: cerebellum posterior lobe
DMN: default mode network
ECN: executive control network
FC: functional connectivity
FCS: functional connectivity strength
FWHM: full-width at half-maximum
GM: gray matter
HCs: healthy controls
IFG: inferior frontal gyrus
LG: lingual gyrus
MCC: middle cingulate cortex
MOG: middle occipital gyrus
MRI: magnetic resonance imaging
NSAIDs: non-steroidal anti-inflammatory drugs
OT: oxytocin
PARCS: predictive and reactive control systems
PCC: posterior cingulate cortex
PCU: precuneus
PDM: primary dysmenorrhea
PGF_2α_: prostaglandin F_2α_
PHG: parahippocampal gyrus
ROI: region of interest
rs-FC: resting-state functional connectivity
rs-FCS: resting-state functional connectivity strength
rs-fMRI: resting-state functional magnetic resonance imaging
VAS: visual analog scale.

## References

[B1] Arsenault J. T. Caspari N. Vandenberghe R. Vanduffel W. (2018). Attention shifts recruit the monkey default mode network. *J. Neurosci.* 38 1202–1217. Epub 2017/12/22. 10.1523/jneurosci.1111-17.2017 29263238 PMC6596261

[B2] Baliki M. N. Geha P. Y. Apkarian A. V. Chialvo D. R. (2008). Beyond feeling: Chronic pain hurts the brain, disrupting the default-mode network dynamics. *J. Neurosci.* 28 1398–1403. Epub 2008/02/08. 10.1523/jneurosci.4123-07.2008 18256259 PMC6671589

[B3] Bansal R. Peterson B. S. (2018). Cluster-level statistical inference in fmri datasets: The unexpected behavior of random fields in high dimensions. *Magn. Reson. Imaging* 49 101–115. Epub 2018/02/07. 10.1016/j.mri.2018.01.004 29408478 PMC5991838

[B4] Baumbach P. MeiSSner W. Reichenbach J. R. Gussew A. (2022). Functional connectivity and neurotransmitter impairments of the salience brain network in chronic low back pain patients: A combined resting-state functional magnetic resonance imaging and 1H-MRS study. *Pain* 10.1097/j.pain.0000000000002626 [Epub ahead of print]. 35417435

[B5] Biggs E. E. Timmers I. Meulders A. Vlaeyen J. W. S. Goebel R. Kaas A. L. (2020). The neural correlates of pain-related fear: A meta-analysis comparing fear conditioning studies using painful and non-painful stimuli. *Neurosci. Biobehav. Rev.* 119 52–65. Epub 2020/10/05. 10.1016/j.neubiorev.2020.09.016 33011229

[B6] Bishop S. J. (2007). Neurocognitive mechanisms of anxiety: An integrative account. *Trends Cogn. Sci.* 11 307–316. Epub 2007/06/08. 10.1016/j.tics.2007.05.008 17553730

[B7] Biswal B. Yetkin F. Z. Haughton V. M. Hyde J. S. (1995). Functional connectivity in the motor cortex of resting human brain using echo-planar mri. *Magn. Reson. Med.* 34 537–541. Epub 1995/10/01. 10.1002/mrm.1910340409 8524021

[B8] Boulloche N. Denuelle M. Payoux P. Fabre N. Trotter Y. Géraud G. (2010). Photophobia in migraine: An interictal pet study of cortical hyperexcitability and its modulation by pain. *J. Neurol. Neurosurg. Psychiatry* 81 978–984. Epub 2010/07/03. 10.1136/jnnp.2009.190223 20595138

[B9] Burnett M. Lemyre M. (2017). No. 345-Primary dysmenorrhea consensus guideline. *J. Obstet. Gynaecol. Can.* 39 585–595. Epub 2017/06/20. 10.1016/j.jogc.2016.12.023 28625286

[B10] Bushnell M. C. Ceko M. Low L. A. (2013). Cognitive and emotional control of pain and its disruption in chronic pain. *Nat. Rev. Neurosci.* 14 502–511. Epub 2013/05/31. 10.1038/nrn3516 23719569 PMC4465351

[B11] Cao Z. M. Chen Y. C. Liu G. Y. Wang X. Shi A. Q. Xu L. F. (2022). Abnormalities of thalamic functional connectivity in patients with migraine: A resting-state fmri study. *Pain Ther.* 11 561–574. Epub 2022/02/28. 10.1007/s40122-022-00365-1 35220550 PMC9098714

[B12] Cauda F. Sacco K. Duca S. Cocito D. D’Agata F. Geminiani G. C. (2009). Altered resting state in diabetic neuropathic pain. *PLoS One* 4:e4542. Epub 2009/02/21. 10.1371/journal.pone.0004542 19229326 PMC2638013

[B13] Dawood M. Y. (2006). Primary dysmenorrhea: Advances in pathogenesis and management. *Obstet. Gynecol.* 108 428–441. Epub 2006/08/02. 10.1097/01.AOG.0000230214.26638.0c 16880317

[B14] Dun W. H. Yang J. Yang L. Ding D. Ma X. Y. Liang F. L. (2017). Abnormal structure and functional connectivity of the anterior insula at pain-free periovulation is associated with perceived pain during menstruation. *Brain Imaging Behav.* 11 1787–1795. Epub 2016/11/11. 10.1007/s11682-016-9646-y 27832449

[B15] Dun W. Fan T. Wang Q. Wang K. Yang J. Li H. (2020). Association between trait empathy and resting brain activity in women with primary dysmenorrhea during the pain and pain-free phases. *Front. Psychiatry* 11:608928. Epub 2020/12/17. 10.3389/fpsyt.2020.608928 33324267 PMC7725799

[B16] Ferries-Rowe E. Corey E. Archer J. S. (2020). Primary dysmenorrhea: Diagnosis and therapy. *Obstet. Gynecol.* 136 1047–1058. Epub 2020/10/09. 10.1097/aog.0000000000004096 33030880

[B17] Fox M. D. Raichle M. E. (2007). Spontaneous fluctuations in brain activity observed with functional magnetic resonance imaging. *Nat. Rev. Neurosci.* 8 700–711. Epub 2007/08/21. 10.1038/nrn2201 17704812

[B18] Friston K. J. (1994). Functional and effective connectivityin neuroimaging: A synthesis. *Hum. brain Mapp.* 2 56–78.

[B19] Fujii T. Okuda J. Tsukiura T. Ohtake H. Miura R. Fukatsu R. (2002). The role of the basal forebrain in episodic memory retrieval: A positron emission tomography study. *NeuroImage* 15 501–508. Epub 2002/02/19. 10.1006/nimg.2001.0995 11848693

[B20] Gou C. Q. Gao J. Wu C. X. Bai D. X. Mou H. Y. Hou X. L. (2016). Moxibustion for primary dysmenorrhea at different interventional times: A systematic review and meta-analysis. *Evidence Based Complement. Alternat. Med.* 2016:6706901. Epub 2017/01/25. 10.1155/2016/6706901 28115970 PMC5225331

[B21] Greicius M. D. Krasnow B. Reiss A. L. Menon V. (2003). Functional connectivity in the resting brain: A network analysis of the default mode hypothesis. *Proc. Natl. Acad. Sci. U.S.A.* 100 253–258. Epub 2002/12/31. 10.1073/pnas.0135058100 12506194 PMC140943

[B22] Hu Q. Li Y. Wu Y. Lin X. Zhao X. (2022). Brain network hierarchy reorganization in Alzheimer’s disease: A resting-state functional magnetic resonance imaging study. *Hum. Brain Mapp.* 43 3498–3507. Epub 2022/04/16. 10.1002/hbm.25863 35426973 PMC9248302

[B23] Iacovides S. Avidon I. Baker F. C. (2015). What we know about primary dysmenorrhea today: A critical review. *Hum. Reprod. Update* 21 762–778. Epub 2015/09/09. 10.1093/humupd/dmv039 26346058

[B24] Ke J. Yu Y. Zhang X. Su Y. Wang X. Hu S. (2020). Functional alterations in the posterior insula and cerebellum in migraine without Aura: A resting-state mri study. *Front. Behav. Neurosci.* 14:567588. Epub 2020/11/03. 10.3389/fnbeh.2020.567588 33132860 PMC7573354

[B25] Kennedy S. (1997). Primary dysmenorrhoea. *Lancet (London, England)* 349:1116. Epub 1997/04/19. 10.1016/s0140-6736(05)63018-8 9113008

[B26] Lefebvre G. Pinsonneault O. Antao V. Black A. Burnett M. Feldman K. (2005). Primary dysmenorrhea consensus guideline. *J. Obstet. Gynaecol. Can.* 27 1117–1146. Epub 2006/03/10. 10.1016/s1701-2163(16)30395-4 16524531

[B27] Li F. Lu L. Shang S. Chen H. Wang P. Muthaiah V. P. (2021). Altered static and dynamic functional network connectivity in post-traumatic headache. *J. Headache Pain* 22:137. Epub 2021/11/15. 10.1186/s10194-021-01348-x 34773973 PMC8590227

[B28] Li H. J. Xu Y. Zhang K. R. Hoptman M. J. Zuo X. N. (2015). Homotopic connectivity in drug-naïve, first-episode, early-onset schizophrenia. *J. Child Psychol. Psychiatry Allied Discip.* 56 432–443. Epub 2014/08/19. 10.1111/jcpp.12307 25130214 PMC4333112

[B29] Li J. Curley W. H. Guerin B. Dougherty D. D. Dalca A. V. Fischl B. (2021). Mapping the subcortical connectivity of the human default mode network. *NeuroImage* 245:118758. Epub 2021/11/29. 10.1016/j.neuroimage.2021.118758 34838949 PMC8945548

[B30] Li T. Zhang S. Ikeda E. Kobinata H. (2022). Functional connectivity modulations during offset analgesia in chronic pain patients: An fmri study. *Brain Imaging Behav.* 16 1794–1802. Epub 2022/03/23. 10.1007/s11682-022-00652-7 35314949

[B31] Li W. L. Liu L. Sun L. H. (2006). [analysis on therapeutic effect of substance-partitioned moxibustion at Guanyuan (Cv 4) and shenque (Cv 8) for treatment of primary dysmenorrhea of cold-damp type]. *Zhongguo Zhen Jiu* 26 481–482. Epub 2006/08/15. 16903598

[B32] Liang B. Lei Z. Yong C. Yang L. Dongya M. Wei L. (2022). Middle cingulate cortex function contributes to response to non-steroidal anti-inflammatory drug in cervical spondylosis patients: A preliminary resting-state fmri study. *Neuroradiology* 64 1401–1410. Epub 2022/04/26. 10.1007/s00234-022-02964-3 35462573

[B33] Liang X. Zou Q. He Y. Yang Y. (2013). Coupling of functional connectivity and regional cerebral blood flow reveals a physiological basis for network hubs of the human brain. *Proc. Natl. Acad. Sci. U.S.A.* 110 1929–1934. Epub 2013/01/16. 10.1073/pnas.1214900110 23319644 PMC3562840

[B34] Liao B. D. Liu Y. E. Peng Z. M. Zhou C. Liu C. He J. J. (2019). [Therapeutic effects on primary dysmenorrhea treated with moxibustion at shenque (Cv 8) and warm needling at guanyuan (Cv 4) and sanyinjiao (Sp 6)]. *Zhongguo Zhen Jiu* 39 367–370. Epub 2019/04/09. 10.13703/j.0255-2930.2019.04.006 30957446

[B35] Liu P. Liu Y. Wang G. Yang X. Jin L. Sun J. (2017). Aberrant default mode network in patients with primary dysmenorrhea: A fmri study. *Brain Imaging Behav.* 11 1479–1485. Epub 2016/10/16. 10.1007/s11682-016-9627-1 27738992

[B36] Maddock R. J. Garrett A. S. Buonocore M. H. (2001). Remembering familiar people: The posterior cingulate cortex and autobiographical memory retrieval. *Neuroscience* 104 667–676. Epub 2001/07/07. 10.1016/s0306-4522(01)00108-7 11440800

[B37] Maguire E. A. Mummery C. J. (1999). Differential modulation of a common memory retrieval network revealed by positron emission tomography. *Hippocampus* 9 54–61. Epub 1999/03/24. 10.1002/(sici)1098-106319999:1<54::Aid-hipo6<3.0.Co;2-o 10088900

[B38] Mao C. P. Yang H. J. Zhang Q. J. Yang Q. X. Li X. H. (2022). Altered effective connectivity within the cingulo-frontal-parietal cognitive attention networks in chronic low back pain: A dynamic causal modeling study. *Brain Imaging Behav.* 16 1516–1527. Epub 2022/01/27. 10.1007/s11682-021-00623-4 35080703

[B39] Miron D. Duncan G. H. Bushnell C. M. (1989). Effects of attention on the intensity and unpleasantness of thermal pain. *Pain* 39 345–352. Epub 1989/12/01. 10.1016/0304-3959(89)90048-1 2616184

[B40] Ning Y. Zheng R. Li K. Zhang Y. Lyu D. Jia H. (2018). The altered granger causality connection among pain-related brain networks in migraine. *Medicine* 97:e0102. Epub 2018/03/09. 10.1097/md.0000000000010102 29517685 PMC5882438

[B41] Oyarzabal E. A. Hsu L. M. Das M. Chao T. H. Zhou J. Song S. (2022). Chemogenetic stimulation of tonic locus coeruleus activity strengthens the default mode network. *Sci. Adv.* 8:eabm9898. Epub 2022/04/30. 10.1126/sciadv.abm9898 35486721 PMC9054017

[B42] Proctor M. L. Murphy P. A. (2016). Herbal and dietary therapies for primary and secondary dysmenorrhoea. *Cochrane Database Syst. Rev.* 3:CD002124. 10.1002/14651858.CD002124 11687013

[B43] Quan S. Yang J. Dun W. Wang K. Liu H. Liu J. (2021). Prediction of pain intensity with uterine morphological features and brain microstructural and functional properties in women with primary dysmenorrhea. *Brain Imaging Behav.* 15 1580–1588. Epub 2020/07/25. 10.1007/s11682-020-00356-w 32705468

[B44] Quevedo A. S. Coghill R. C. (2007). Attentional modulation of spatial integration of pain: Evidence for dynamic spatial tuning. *J. Neurosci.* 27 11635–11640. Epub 2007/10/26. 10.1523/jneurosci.3356-07.2007 17959806 PMC6673211

[B45] Saykin A. J. Johnson S. C. Flashman L. A. McAllister T. W. Sparling M. Darcey T. M. (1999). Functional differentiation of medial temporal and frontal regions involved in processing novel and familiar words: An fmri study. *Brain J. Neurol.* 122(Pt 10), 1963–1971. Epub 1999/10/03. 10.1093/brain/122.10.1963 10506097

[B46] Schlund M. W. Brewer A. T. Magee S. K. Richman D. M. Solomon S. Ludlum M. (2016). The tipping point: Value differences and parallel dorsal-ventral frontal circuits gating human approach-avoidance behavior. *NeuroImage* 136 94–105. Epub 2016/05/08. 10.1016/j.neuroimage.2016.04.070 27153979

[B47] She Y. F. Sun L. H. Yang J. J. Ge J. J. Li X. H. Lu Y. J. (2008). [Effects of substance-partitioned moxibustion on plasma Beta-Ep content in the patient with primary dysmenorrhea of cold-damp stagnation type in the menstrual period]. *Zhongguo Zhen Jiu* 28 719–721. Epub 2008/11/01. 18972726

[B48] Shen Z. Yu S. Wang M. She T. Yang Y. Wang Y. (2019). Abnormal amygdala resting-state functional connectivity in primary dysmenorrhea. *Neuroreport* 30 363–368. Epub 2019/02/15. 10.1097/wnr.0000000000001208 30762615

[B49] Sun L. H. Ge J. J. Yang J. J. She Y. F. Li W. L. Li X. H. (2009). [Randomized controlled clinical study on ginger-partitioned moxibustion for patients with cold-damp stagnation type primary dysmenorrhea]. *Zhen Ci Yan Jiu* 34 398–402. Epub 2010/03/10. 20209976

[B50] Tanglay O. Young I. M. Dadario N. B. Briggs R. G. Fonseka R. D. Dhanaraj V. (2022). Anatomy and white-matter connections of the precuneus. *Brain Imaging Behav.* 16 574–586. Epub 2021/08/28. 10.1007/s11682-021-00529-1 34448064

[B51] Tops M. Boksem M. A. Quirin M. (2014). H IJ, Koole SL. Internally directed cognition and mindfulness: An integrative perspective derived from predictive and reactive control systems theory. *Front. Psychol.* 5:429. Epub 2014/06/07. 10.3389/fpsyg.2014.00429 24904455 PMC4033157

[B52] Uddin L. Q. Kelly A. M. Biswal B. B. Castellanos F. X. Milham M. P. (2009). Functional connectivity of default mode network components: Correlation, anticorrelation, and causality. *Hum. Brain Mapp.* 30 625–637. Epub 2008/01/26. 10.1002/hbm.20531 18219617 PMC3654104

[B53] Villemure C. Slotnick B. M. Bushnell M. C. (2003). Effects of odors on pain perception: Deciphering the roles of emotion and attention. *Pain* 106 101–108. Epub 2003/10/29. 10.1016/s0304-3959(03)00297-5 14581116

[B54] Wang S. M. Li X. G. Zhang L. Q. Xu Y. C. Li Q. (2005). [Clinical observation on medicine-separated moxibustion for treatment of primary dysmenorrhea and study on the mechanism]. *Zhongguo Zhen Jiu* 25 773–775. Epub 2005/12/13. 16335202

[B55] Wang Y. Li Y. Ma X. Chen S. Peng Y. Hu G. (2022). Regional homogeneity alterations in patients with impaired consciousness. An observational resting-state fmri study. *Neuroradiology* 64 1391–1399. Epub 2022/02/03. 10.1007/s00234-022-02911-2 35107592

[B56] Wei X. Shi G. Tu J. Zhou H. Duan Y. Lee C. K. (2022). Structural and functional asymmetry in precentral and postcentral gyrus in patients with unilateral chronic shoulder pain. *Front. Neurol.* 13:792695. Epub 2022/03/08. 10.3389/fneur.2022.792695 35250808 PMC8892006

[B57] Wiech K. Ploner M. Tracey I. (2008). Neurocognitive aspects of pain perception. *Trends Cogn. Sci.* 12 306–313. Epub 2008/07/09. 10.1016/j.tics.2008.05.005 18606561

[B58] Xue C. Qi W. Yuan Q. Hu G. Ge H. Rao J. (2021). Disrupted dynamic functional connectivity in distinguishing subjective cognitive decline and amnestic mild cognitive impairment based on the triple-network model. *Front. Aging Neurosci.* 13:711009. Epub 2021/10/05. 10.3389/fnagi.2021.711009 34603006 PMC8484524

[B59] Yang J. J. Sun L. H. She Y. F. Ge J. J. Li X. H. Zhang R. J. (2008). [Influence of ginger-partitioned moxibustion on serum no and plasma endothelin-1 contents in patients with primary dysmenorrhea of cold-damp stagnation type]. *Zhen Ci Yan Jiu* 33 409–412. Epub 2009/03/18. 19288904

[B60] Yang L. Dun W. Li K. Yang J. Wang K. Liu H. (2019). Altered amygdalar volume and functional connectivity in primary dysmenorrhoea during the menstrual cycle. *Eur. J. Pain (London, England)* 23 994–1005. Epub 2019/01/22. 10.1002/ejp.1368 30664322

[B61] Yang M. Chen X. Bo L. Lao L. Chen J. Yu S. (2017). Moxibustion for pain relief in patients with primary dysmenorrhea: A randomized controlled trial. *PloS One* 12:e0170952. Epub 2017/02/09. 10.1371/journal.pone.0170952 28170396 PMC5295763

[B62] Yang M. He H. Duan M. Chen X. Chang X. Lai Y. (2018). The effects of music intervention on functional connectivity strength of the brain in schizophrenia. *Neural Plast.* 2018:2821832. Epub 2018/06/02. 10.1155/2018/2821832 29853841 PMC5954893

[B63] Yesuf T. A. Eshete N. A. Sisay E. A. (2018). Dysmenorrhea among university health science students, Northern Ethiopia: Impact and associated factors. *Int. J. Reprod. Med.* 2018:9730328. Epub 2018/04/04. 10.1155/2018/9730328 29610764 PMC5828460

[B64] Zahradnik H. P. Hanjalic-Beck A. Groth K. (2010). Nonsteroidal anti-inflammatory drugs and hormonal contraceptives for pain relief from dysmenorrhea: A review. *Contraception* 81 185–196. Epub 2010/02/18. 10.1016/j.contraception.2009.09.014 20159173

[B65] Zhang Y. N. Huang Y. R. Liu J. L. Zhang F. Q. Zhang B. Y. Wu J. C. (2020). Aberrant resting-state cerebral blood flow and its connectivity in primary dysmenorrhea on arterial spin labeling mri. *Magn. Reson. imaging* 73 84–90. Epub 2020/08/05. 10.1016/j.mri.2020.07.012 32750444

[B66] Zhang Y. Huang Y. Liu N. Wang Z. Wu J. Li W. (2022). Abnormal interhemispheric functional connectivity in patients with primary dysmenorrhea: A resting-state functional mri study. *Quant. Imaging Med. Surg.* 12 1958–1967. Epub 2022/03/15. 10.21037/qims-21-731 35284283 PMC8899927

[B67] Zhu Y. Chen R. L. Le J. I. Miao F. R. (2010). [Efficacy observation of primary dysmenorrhea treated with isolated-herbal moxibustion on shenque (Cv 8)]. *Zhongguo Zhen Jiu* 30 453–455. Epub 2010/06/29. 20578380

[B68] Zou Y. Tang W. Qiao X. Li J. (2021). Aberrant modulations of static functional connectivity and dynamic functional network connectivity in chronic migraine. *Quant. Imaging Med. Surg.* 11 2253–2264. Epub 2021/06/04. 10.21037/qims-20-588. 34079699 PMC8107335

[B69] Zuo L. Zhou Y. Wang S. Wang B. Gu H. Chen J. (2019). Abnormal brain functional connectivity strength in the overactive bladder syndrome: A resting-state fmri study. *Urology* 131 64–70. Epub 2019/06/01. 10.1016/j.urology.2019.05.019 31150692

[B70] Zuo X. Ling Y. Jackson T. (2022). Testing links between pain-related biases in visual attention and recognition memory: An eye-tracking study based on an impending pain paradigm. *Q. J. Exp. Psychol.* (Hove) 17470218221102922. 10.1177/17470218221102922 [Epub ahead of print]. 35570662

